# Somatic health among heroin addicts before and during opioid maintenance treatment: a retrospective cohort study

**DOI:** 10.1186/1471-2458-8-43

**Published:** 2008-01-31

**Authors:** Ivar Skeie, Mette Brekke, Morten Lindbæk, Helge Waal

**Affiliations:** 1 Aker University Hospital, Oslo, Norway; 2 University of Oslo, Faculty of Medicine, Institute of Psychiatry, Norwegian Centre for Addiction Research, Oslo, Norway; 3 University of Oslo, Faculty of Medicine, Institute of General Practice and Community Medicine, Oslo, Norway

## Abstract

**Background:**

The long-term impact of opioid maintenance treatment (OMT) on morbidity and health care utilization among heroin addicts has been insufficiently studied. The objective of this study was to investigate whether health care utilization due to somatic disease decreased during OMT, and if so, whether the reduction included all kinds of diseases and whether a reduction was related to abstinence from drug use.

**Methods:**

Cohort study with retrospective registration of somatic disease incidents (health problems, acute or sub-acute, or acute problems related to chronic disease, resulting in a health care contact). Medical record data were collected from hospitals, Outpatients' Departments, emergency wards and from general practitioners (GPs) and prospective data on substance use during OMT were available from 2001 onwards. The observation period was five years before and up to five years during OMT. The cohort consisted of 35 out of 40 patients who received OMT between April 1999 and January 2005 in a Norwegian district town. Statistical significance concerning changes in number of incidents and inpatient and outpatient days during OMT compared with the pre OMT period was calculated according to Wilcoxon signed rank test. Significance concerning pre/during OMT changes in disease incidents by relation to the type of health service contacts, as well as the impact of ongoing substance use during OMT on the volume of contacts, was calculated according to Pearson chi-square and Fisher's exact tests.

**Results:**

278 disease incidents were registered. There was a reduction in all incidents by 35% (p = 0.004), in substance-related incidents by 62% (p < 0.001) and in injection-related incidents by 70% (p < 0.001). There was an insignificant reduction in non-fatal overdose incidents by 44% (p = 0.127) and an insignificant increase in non-substance-related incidents by 13% (p = 0.741). Inpatient and outpatient days were reduced by 76% (p = 0.003) and 46% (p = 0.060), respectively. The disease incidents were less often drug-related during OMT (p < 0.001). Patients experienced a reduction in substance-related disease incidents regardless of ongoing substance use, however there was a trend towards greater reductions in those without ongoing abuse.

**Conclusion:**

Although as few as 35 patients were included, this study demonstrates a significant reduction in health care utilization due to somatic disease incidents during OMT. The reduction was most pronounced for incidents related to substance use and injection. Inpatient and outpatient days were reduced. Most probably these findings reflect somatic health improvement among heroin addicts during OMT.

## Background

Opioid addicts, especially injecting heroin users, suffer increased health problems [1-3] and reduced health related quality of life (HRQOL) [4-7] as well as increased mortality, compared to the general population [8-10]. This is particularly related to overdoses [11-14], injuries [9], human immunodeficiency virus (HIV)-infection [9,11], viral hepatitis B (HBV) [15,16] and viral hepatitis C (HCV) [16,17] with end-state liver disease and other infections like endocarditis [9], osteomyelitis [18] and others [19,20]. Several studies and case reports demonstrate vulnerability among injecting drug users (IDUs) to rare infectious diseases like tetanus [21], botulism [22,23] and gas gangrene due to Clostridium [21,24-27]. Increased prevalence of various psychiatric diseases among substance users is well documented in population surveys and among persons entering opioid maintenance treatment (OMT) [28-33].

In spite of considerable morbidity, drug users frequently neglect their health problems, and diseases may remain untreated. Several studies describe that patients with extensive drug use cause problems in hospitals [34] and are difficult to treat in ordinary general practice. Yet some studies based on central health registers show increased health care utilization, in particular due to intoxications/overdoses, infections related to illicit drug use and injuries [35].

OMT leads to reduced illegal opioid use and injection [36-39] which probably reduces overdoses and infections. It is also likely that OMT improves nutritional status and general health. Moreover, OMT patients may become more motivated to seek medical help, and OMT may remove or at least reduce tension between patients and health service providers, thus leading to improved health care follow-up. It is therefore reasonable to assume that over time OMT will reduce morbidity and mortality. Reduction in mortality during OMT has been shown in observational studies [11], but in two recent meta-analyzes of randomised controlled trials (RCTs) mortality reduction could not be documented [36,38]. However, this might be due to problems applying RCT design in studies on OMT effects versus placebo or no treatment [40]. Some studies have shown improved psychological well-being, reduced frequency of self-reported physical health problems, and improved self-perceived HRQOL during OMT [37,41]. Except for this, documentation of OMT-induced health effects is poor [42]. Consequently observational studies with careful design might increase the understanding of OMT effects on health care utilization and also on morbidity.

With a national OMT program implemented during a short time span and a well documented and severe illicit drug problem [39], Norway is well suited for such studies. The number of IDUs in Norway is estimated to 8 200 – 12 500 persons out of a population of 4.7 millions in 2005 [43]. The number of OMT patients December 31^th ^2005 was 3 614 [44]. Although heroin is usually injected [45] the prevalence of HIV among heroin users is as low as 1–2% [46]. The cumulative number of IDUs infected with HIV from the early 1980s until 2006 is 528 [46]. The anti-HCV antibody prevalence among IDUs is 70–80% [47] and approximately 2/3 of these are Polymerase Chain Reaction (PCR) positive [48]. The death rate among drug users has been estimated to about 1–2% per year [45]. The number of registered overdose deaths has been high with a peak of 405 in 2001 falling to 231 in 2004 [45]. Severe psychiatric co-morbidity ("double-diagnosis") is documented in about 1/3 of IDUs [49].

The national OMT program keeps overall surveillance of patients entering and leaving. Entrance depends on specific criteria [39]: patients should be at least 25 years old, have been dependent on opioids for "several" years and have undergone abstinence-oriented treatment. Treatment is ended if patients fail to pick up the medication over time, and may be involuntary terminated if patients show continued illicit drug use, sell the OMT-medication or illegal drugs, act threatening or violent towards treatment personnel or show severe lack of willingness to fulfil the program regulations. Authorized regional centres cooperate with municipal social service and GPs. Only methadone and buprenorphine are accepted as substitution medication and the average dosage level is high: 114 mg and 18 mg respectively in 2005 [44]. Retention in treatment – which means the proportion of patients who stay in the program over time – is high, compared to most other countries [39].

The systematic collection of information on all participants in the OMT-program as well as computer-based record systems in primary health care and hospitals make Norway suitable for detailed studies of OMT related health effects. The objective of this study was to investigate health care utilization due to somatic disease before versus during OMT in a cohort of OMT-patients. The hypothesis was that such health care contact would decrease during OMT, mainly due to reduced health problems related to illicit drug use and injection. Further, we wanted to investigate whether such possible reduction would occur only in patients who stayed abstinent from illicit drug use or also among those with ongoing abuse.

## Methods

Our study compares health care utilization due to somatic disease before versus during OMT using a retrospective cohort design.

### Study population

The study was carried out in Gjoevik, a district town with 28 000 inhabitants and with considerable drug problems. OMT was started locally in 1999, according to the guidelines of the national program. However, over the years the GPs have come to play a more important role than is typical for OMT in Norway. Further, very few patients, even among those with ongoing substance use, have had their treatment involuntarily terminated, rather they have received increased follow-up by GPs and social workers. Outcome concerning social rehabilitation and continued substance use during OMT has been close to national average [50]. By the end of 2005, all 40 patients who had started OMT were still in treatment, and 36 consented to participate in the study. Data were not collected for one person, rendering 35 participants (87.5%). Key characteristics of the study population are summarized in Table 1.

**Table 1:** Cohort characteristics

	**Male**	**Female**
	
Gender, n (%)	22 (63)	13 (37)
Age at OMT-start, years, mean (range)	37.3 (29.4 – 50.5)	37.5 (27.3 – 50.3)
OMT medication, methadone, n	19	11
OMT medication, buprenorphine, n	3	2
Methadone dosage mg, median (range)	132.5 (100 – 220)	145 (100 – 170)*
Buprenorphine dosage mg, median (range)	22(16 – 32)	22 (20 – 24)
HCV antibody positive, n (%)	21 (95.5)	13 (100)
Receiving anti HCV treatment during OMT, n	1	0
HIV antibody positive, n	0	0
Died during OMT, n	0	0

*One outlier, 580 mg

The observation period was five years prior to and up to five years during OMT; the mean observation period during OMT was 35 months. Three patients temporarily terminated OMT and then restarted. Disease incidents and health care utilization that occurred while the three patients were between OMT periods (in total five years) were counted as pre-OMT.

### Data sources

Thirty-two of the participants were interviewed about disease incidents during OMT and the years prior to OMT. One of the authors (IS, physician) performed all interviews, which took place in a primary care centre or in the patient's home. As no validated questionnaire suitable for collecting this information was available, a list of relevant diagnoses (Table 2) was used, as well as Time-line Follow-back procedures, in order to facilitate remembering disease incidents and treatment.

**Table 2:** Before/during OMT changes in disease incidents and inpatient and outpatient days. Number of somatic disease incidents* and inpatient and outpatient days* per 100 patient years before and during opioid maintenance treatment (OMT) in 35 patients.

	Before OMT	During OMT	Reduction %	Increase %	P-value**
	Incidents/treatment days per 100 patient ears	Incidents/treatment days per 100 patient years			
**Substance-related incidents**					
Overdoses (non-fatal)	17.7	9.9	44		0.127
Injection-related incidents, total***	40.6	11.8	70		<0.001
*Acute thromboembolic incidents*	*4.6*	*0.0*			
*Acute hepatitis*	*1.7*	*0.0*			
*Acute local infection*	*32.6*	*10.9*			
*Acute/sub-acute general infection*	*1.7*	*1.0*			
Other substance-related incidents	21.7	8.9	59		0.087

Total	80.0	30.6	62		<0.001

**Non-substance-related incidents**					
Infections	10.3	8.9	14		0.849
Injuries	20.6	19.7	4		0.832
Other incidents	12.6	20.7		64	0.375

Total	43.5	49.3		13	0.741

**All incidents**	123.5	79.9	35		0.004

**Treatment days**					
Inpatient days	257.0	61.0	76		0.003
Outpatient days	59.0	32.0	45		0.060

*Definition of disease incident and treatment days, see text

**Wilcoxon signed rank test

***Overdoses not included, subcategories of incidents in italic

Based upon the information obtained in the interviews, records from hospitals, emergency wards and GPs were collected. For the three persons not interviewed, hospital records were collected based upon information in their GPs' records. All requested records concerning inpatient treatment, treatment in Outpatients' Departments (in Norway these are hospital units), emergency wards (in Norway these are part of the primary health care and staffed by GPs), and 75 out of 82 records from solo GPs and GP groups (in Norway most GPs work together in groups of 3–5 sharing a joint record system) were received and scrutinized. Data collection was concluded in June 2005. All data on diagnosis and health care utilization presented in the study originate from these records. Admissions and health care visits mentioned by patients which could not be verified from records were not included. Records from hospitals and GPs which had not been specified by the participants were not requested.

### Measures

A "disease incident" was defined as a health problem, acute or sub-acute, resulting in a health care contact. Only somatic incidents were counted, psychiatric illness was only considered if it caused a somatic incident, e.g. an injury due to self harm. A disease incident could be an isolated case, for instance an overdose, an infection or an injury, or a new incident due to an underlying chronic disease, for instance an asthma attack. Even if a disease incident lead to more than one health care visit, e.g. follow-up visits for a fracture, it was registered as one incident. Routine hospital or GP check ups for chronic diseases or repeated treatment visits for a chronic disease, e.g. hepatitis C, were not included. Disease incidents documented in several records, e.g. from a hospital and a GP, were only counted once. We also counted number of inpatient treatment days (inpatient days) and treatment days in hospitals' Outpatients' Departments (outpatient days) due to the disease incidents we registered.

The full-text records were scrutinized by one of the authors (IS). ICD-10 [51] diagnoses from hospitals and ICPC [52] diagnoses from GPs were registered. Based on record information the disease incidents were categorized by mean of a diagnosis list developed for this study (Table 2). The list differentiates between drug related incidents and others. Drug related incidents were categorized as overdoses, injection related incidents and "others", like rhabdomyolysis and related neuro-muscular damage related to non-fatal overdoses, severe withdrawal reactions, inpatient treatment because of severe exhaustion, malnutrition and poor general condition due to drug use, severe sub-acute dental health problems and several others. The incidents not related to drug use were divided into infections, injuries and "others", the latter including all incidents not fitting into the specific categories.

Inter-rater agreement on relation to substance use and diagnostic categories was estimated for 22 disease incidents in six patients by two independent investigators (IS and another physician). Agreement regarding relation to substance use was perfect with a kappa value (κ) of 1. When diagnostic groups were considered, κ was 0.82.

Information about ongoing use of illicit drugs and alcohol during OMT, based on urinary testing and clinical assessment, was gathered from the annual reports made for each OMT patient in Norway since 2001 [39]. For four patients the treatment period was too short or provided insufficient information on substance use; thus rendering such information for 31 patients. The annual report scores overall drug use during the last four weeks on a five-point scale. In our study we simplified this to a dichotomized score for the entire treatment period, differentiating between "problematic" use with severe consequences for psychosocial function versus "abstinence or non-problematic use" without such consequences.

### Statistics and ethics

Wilcoxon signed rank test was used to compare changes in rates of episodes before versus during OMT. Pearson chi-square and Fisher's exact test were used to evaluate the changes in the proportion of incidents related to substance use as well as assessment of health improvement versus ongoing use of illegal drugs and alcohol during OMT. Inter-rater agreement was estimated according to Cohen's kappa. All statistical calculations were performed in SPSS 14.0.

The Regional Committee for Research Ethics approved the study.

## Results

Table 1 gives a summary of basic demographic and treatment characteristics for the patient sample. The gender distribution is typical for IDUs and mean age at OMT start is 37 years. Treatment is high dosage, dominantly with methadone as agonist. Nearly all patients are HCV-antibody positive, reflecting the dominant injecting drug use pattern.

Altogether, 278 disease incidents were registered during the observation period, 197 before and 81 during OMT.

Table 2 presents findings on health care utilization before and during OMT. The overall reduction in disease incidents was 35% (p = 0,004). There was a reduction of 62% in substance-related incidents (p < 0.001), a 70% reduction in injection related incidents (p < 0.001), and an insignificant reduction of 44 and 59% respectively in overdoses and other substance-related incidents. There was an insignificant increase of 13% in non-substance-related disease incidents, exclusively in the group "other", while infections and injuries showed minor change. Inpatient and outpatient days due to somatic disease incidents were reduced by 76% (p = 0.003) and 46% (p = 0.060) respectively.

Table 3 shows the pre/during OMT shift in the distribution of disease incidents by relation to substance use. Before OMT 62% of the incidents were related to substance use, compared to 36% during OMT (p < 0.001).

**Table 3:** Distribution of somatic disease incidents before and during OMT by relation to substance use. N = 278.

Relation to substance use*	Before OMT (%)	During OMT (%)	P-value**
*Related*	*123 (62)*	*29 (36)*	
*Not related*	*74 (38)*	*52 (64)*	

Total	197 (100)	81 (100)	<0.001

* Inter-rater agreement κ = 1.00

**Pearson chi-square test

Table 4 displays health service contacts made during the 278 disease incidents. Forty per cent of all disease incidents during OMT were documented exclusively by GPs, compared with 25% before OMT (p = 0.02). Around 90% of all hospital treatment, before as well as during OMT, took place at the local hospital in Gjoevik.

**Table 4:** Changes in type of health service contact. Before/during OMT changes in distribution of somatic disease incidents separated by type of health service contact. N = 278

	Number of incidents (%)	
	GP*	Hosp+**

Before OMT	49 (25)	148 (75)
During OMT	32 (40)	49 (60)

P-value	0.015***	

*General practitioner

**Hospital/outpatient clinic/emergency ward

*** Pearson chi-square test

Table 5 shows changes in disease incidents in nine patients with and 22 patients without problematic substance use during OMT. Regarding injection-related incidents, there was no difference between the groups, both showing improvement. The reduction in all substance-related incidents was greater for patients without problematic drug use, but the difference was not statistically significant (p = 0.06). The reduction in the total number of incidents was significantly greater for patients without problematic drug use (p = 0.007).

**Table 5:** Health care utilization versus ongoing illicit drug use during OMT. Number of patients with reduced, unchanged or increased rates of all, substance-related and injection-related somatic disease incidents respectively, in 22 patients with and 9 patients without problematic* illicit drug use during OMT

Diagnose group	Change in incidents during versus before OMT, number of patients				
	
*Illicit drug use*	Reduction	Unchanged	Increase	Total	P value**
All incidents					0.007
*Abstinence or non-problematic*	*18*	1***	*3*	*22*	
*Problematic*	*3*	*0*	*6*	*9*	

All substance-related incidents					0.063
*Abstinence or non-problematic*	*18*	3****	*1*	*22*	
*Problematic*	*6*	*0*	*3*	*9*	

Injection-related incidents					0.503
*Abstinence or non-problematic*	*15*	6***	*1*	*22*	
*Problematic*	*6*	2***	*1*	*9*	

* Definition of problematic drug use, see text

**Chi-square Fisher's exact test: number of patients with increased versus reduced/unchanged rates of all, substance-related and injection-related incidents respectively, versus illicit drug use during OMT

*** Patients had zero episodes during both time periods

****Two of the three patients had zero episodes

## Discussion

The primary goal of this study was to investigate how OMT influences health service utilization in heroin addicts. The study demonstrates a significant reduction in health care contacts due to somatic disease incidents during the five first years of OMT compared to the five previous years. This is a significant finding. Even if several studies have shown severe morbidity among heroin addicts, and some have found health improvement during maintenance treatment [37,41], we have not been able to find any study systematically comparing somatic morbidity before OMT with morbidity during treatment, based on record information.

The key question regarding the interpretation of our findings is whether the observed reduction in health care utilization can be seen as an indicator of health improvement during OMT compared to the period before. Firstly, how complete was the registration of admissions and health care visits? The study cohort includes nearly all OMT-patients in a defined area; hence selection bias was not a problem. Recall bias could be a problem, greater the further back we go. The patients' information turned out to be chiefly correct, when controlled against the records, regarding type of disease or injury and where treatment had been received, but more imprecise regarding the point of time. Each patient had on average been treated at two GP centres, and approximately 90% of all hospital treatment had taken place at the local hospital which shows a high degree of stability in the relation between treatment services and the patient group in Gjoevik. The study thus comprises the majority of health service contacts due to somatic disease incidents during the study period.

Secondly, there will be a gap between the volume of disease in any patient sample, and what results in health service contacts, and this is particularly so in a population of IDUs [34]. Due to the structure of the treatment program, contact between patients and the health services was close during OMT, probably leading to increased help-seeking and better medical follow-up and tending to reduce the proportion of disease incidents not resulting in a health service contact. Thirdly, the patients were five years older during the OMT period, leading to increased somatic morbidity. These factors all tend to increase the volume of registered health care contacts during OMT. Hence, when our study still shows a decline in utilization of health services, this most probably is a proxy for an improvement in somatic health status, and moreover, the OMT-induced improvement is probably more extensive than our findings indicate.

Even six out of nine patients with ongoing problematic substance use during OMT experienced a reduction in drug related disease incidents. The most likely explanation is that they stop or at least reduce injecting drugs. However, due to the increase in non-substance-related disease incidents, the majority of patients with problematic substance-use showed an increase in the total number of incidents during OMT. This could be a consequence of changed help-seeking behaviour and better medical follow-up during OMT. If so, this finding reflects improved follow-up and not a true rise in morbidity. On the other hand, it is conceivable that patients with ongoing drug abuse during OMT are more exposed to disease than those without. However, because of the small number of patients, and some uncertainty concerning the differentiation between patients with and without ongoing problematic substance abuse, these results and their significance should be interpreted with caution.

OMT is often evaluated primarily by its effect on social rehabilitation and continued substance use. According to our findings, this is not sufficient. Drug related disease incidents were reduced even among patients with ongoing abuse, though to a lesser degree. This might question involuntary termination of OMT in patients who still take illegal drugs.

The study has some weaknesses. The cohort is small and limited to one local community. The research instruments, especially the diagnosis categorisation system, have not been validated by other researchers. In addition, it is not always obvious whether a disease incident is related to substance use or not. However, the high level of inter-rater agreement on whether incidents were substance related or not (κ = 1) implies that this is possible to differentiate.

In spite of these weaknesses, our study of a small patient cohort showed a significant reduction in health care contacts caused by somatic disease incidents during OMT compared to the five years prior to treatment. These findings ought to be further investigated in an enlarged study. This could bring information about factors influencing somatic health status changes during OMT, like psychiatric co-morbidity or living in a larger city. The design chosen appears suitable for investigating OMT-related changes in somatic morbidity among heroin addicts in Norway.

## Conclusion

Even with as few as 35 patients included, this study demonstrates a significant decrease in health care contacts due to somatic disease incidents during OMT compared to the five years before entering treatment. This reduction was most striking for incidents related to substance use, and drug injection in particular. Inpatient treatment days and treatment days in hospitals' Outpatients' Departments were reduced during OMT. These findings most probably reflect an improvement in somatic health status for drug abusers undergoing OMT compared to the period before entering treatment.

## Abbreviations

GP – general practitioner (physician)

HBV – hepatitis B virus

HCV – hepatitis C virus

HIV, human immunodeficiency virus

HRQOL, health related quality of life

IDU, injecting drug user

OMT, opioid maintenance treatment

PCR, Polymerase chain reaction

RCT, randomised controlled trial

SPSS, Statistical Package for the Social Sciences

## Competing interests

The authors declare that they have no competing interests.

## Authors' contributions

IS had the original idea for the study, participated in the planning, carried out the collection of data, performed the statistical analysis, drafted the manuscript and is the primary author of the paper. HW was project leader, main supervisor and participated in the planning of the study and the writing of the article. MB was supervisor and participated in the planning of the study and the writing of the article. ML was supervisor and participated in the planning of the study, the statistical analysis and the writing of the article. All authors read and approved the final manuscript.

## Pre-publication history

The pre-publication history for this paper can be accessed here:



## References

[B1] Fischer B, Rehm J, Brissette S, Brochu S, Bruneau J, El-Guebaly N, Noel L, Tyndall M, Wild C, Mun P, Baliunas D (2005). Illicit opioid use in Canada: comparing social, health, and drug use characteristics of untreated users in five cities (OPICAN study). J Urban Health.

[B2] Gjeruldsen SR, Myrvang B, Opjordsmoen S (2003). A 25-year follow-up study of drug addicts hospitalised for acute hepatitis: present and past morbidity. European addiction research.

[B3] Ross J, Teesson M, Darke S, Lynskey M, Ali R, Ritter A, Cooke R (2005). The characteristics of heroin users entering treatment: findings from the Australian treatment outcome study (ATOS). Drug Alcohol Rev.

[B4] Dalgard O, Egeland A, Skaug K, Vilimas K, Steen T (2004). Health-related quality of life in active injecting drug users with and without chronic hepatitis C virus infection. Hepatology (Baltimore, Md.

[B5] Millson P, Challacombe L, Villeneuve PJ, Strike CJ, Fischer B, Myers T, Shore R, Hopkins S (2006). Determinants of health-related quality of life of opiate users at entry to low-threshold methadone programs. European addiction research.

[B6] Millson PE, Challacombe L, Villeneuve PJ, Fischer B, Strike CJ, Myers T, Shore R, Hopkins S, Raftis S, Pearson M (2004). Self-perceived health among Canadian opiate users: a comparison to the general population and to other chronic disease populations. Canadian journal of public health.

[B7] Puigdollers E, Domingo-Salvany A, Brugal MT, Torrens M, Alvaros J, Castillo C, Magri N, Martin S, Vazquez JM (2004). Characteristics of heroin addicts entering methadone maintenance treatment: quality of life and gender. Substance use & misuse.

[B8] Bargagli AM, Hickman M, Davoli M, Perucci CA, Schifano P, Buster M, Brugal T, Vicente J (2006). Drug-related mortality and its impact on adult mortality in eight European countries. European journal of public health.

[B9] Darke S, Degenhardt L, Mattick R, Griffith Edwards (2007). Mortality Amongst Illicit Drug Users: Epidemiology, Causes and Intervention. International research monographs in the addictions (IRMA).

[B10] Pasarin MI, Borrell C, Brugal MT, Diaz-Quijano E (2004). Weighing social and economic determinants related to inequalities in mortality. J Urban Health.

[B11] Brugal MT, Domingo-Salvany A, Puig R, Barrio G, Garcia de Olalla P, de la Fuente L (2005). Evaluating the impact of methadone maintenance programmes on mortality due to overdose and aids in a cohort of heroin users in Spain. Addiction (Abingdon, England).

[B12] Darke S (2003). Polydrug use and overdose: overthrowing old myths. Addiction (Abingdon, England).

[B13] Darke S, Hall W (2003). Heroin overdose: research and evidence-based intervention. J Urban Health.

[B14] Steentoft A, Teige B, Holmgren P, Vuori E, Kristinsson J, Hansen AC, Ceder G, Wethe G, Rollmann D (2006). Fatal poisoning in Nordic drug addicts in 2002. Forensic science international.

[B15] Gjeruldsen S, Myrvang B (2002). Hepatitis B virus infection in drug addicts: no acute fatalities, no chronicity and could have benefits. Apmis.

[B16] Reimer J, Lorenzen J, Baetz B, Fischer B, Rehm J, Haasen C, Backmund M (2007). Multiple viral hepatitis in injection drug users and associated risk factors. J Gastroenterol Hepatol.

[B17] Taylor A, Goldberg D, Hutchinson S, Cameron S, Gore SM, McMenamin J, Green S, Pithie A, Fox R (2000). Prevalence of hepatitis C virus infection among injecting drug users in Glasgow 1990-1996: are current harm reduction strategies working?. The Journal of infection.

[B18] Fox IM, Brady K (1997). Acute hematogenous osteomyelitis in intravenous drug users. J Foot Ankle Surg.

[B19] Kimura AC, Higa JI, Levin RM, Simpson G, Vargas Y, Vugia DJ (2004). Outbreak of necrotizing fasciitis due to Clostridium sordellii among black-tar heroin users. Clin Infect Dis.

[B20] Sierra JM, Sanchez F, Castro P, Salvado M, de la Red G, Libois A, Almela M, March F, Espanol M, Sambeat MA, Romeu J, Brugal MT, Garcia de Olalla P, Gatell JM, Vila J, Garcia F, Lopez Colomes JL, Cayla JA, Coll P (2006). Group A streptococcal infections in injection drug users in Barcelona, Spain: epidemiologic, clinical, and microbiologic analysis of 3 clusters of cases from 2000 to 2003. Medicine.

[B21] Brett MM, Hood J, Brazier JS, Duerden BI, Hahné SJ (2005). Soft tissue infections caused by spore-forming bacteria in injecting drug users in the United Kingdom. Epidemiol Infect.

[B22] Akbulut D, Dennis J, Gent M, Grant KA, Hope V, Ohai C, McLauchlin J, Mithani V, Mpamugo O, Ncube F, de Souza-Thomas L (2005). Wound botulism in injectors of drugs: upsurge in cases in England during 2004. Euro Surveill.

[B23] Galldiks N, Nolden-Hoverath S, Kosinski CM, Stegelmeyer U, Schmidt S, Dohmen C, Kuhn J, Gerbershagen K, Bewermeyer H, Walger P, Biniek R, Neveling M, Jacobs AH, Haupt WF (2007). Rapid geographical clustering of wound botulism in Germany after subcutaneous and intramuscular injection of heroin. Neurocritical care.

[B24] Brown PD, Ebright JR (2002). Skin and Soft Tissue Infections in Injection Drug Users. Curr Infect Dis Rep.

[B25] Ringertz SH, Hoiby EA, Jensenius M, Maehlen J, Caugant DA, Myklebust A, Fossum K (2000). Injectional anthrax in a heroin skin-popper. Lancet.

[B26] Taylor A, Hutchinson S, Lingappa J, Wadd S, Ahmed S, Gruer L, Taylor TH, Roy K, Gilchrist G, McGuigan C, Penrice G, Goldberg D (2005). Severe illness and death among injecting drug users in Scotland: a case-control study. Epidemiol Infect.

[B27] McGuigan CC, Penrice GM, Gruer L, Ahmed S, Goldberg D, Black M, Salmon JE, Hood J (2002). Lethal outbreak of infection with Clostridium novyi type A and other spore-forming organisms in Scottish injecting drug users. Journal of medical microbiology.

[B28] Bijl RV, Ravelli A, van Zessen G (1998). Prevalence of psychiatric disorder in the general population: results of The Netherlands Mental Health Survey and Incidence Study (NEMESIS). Soc Psychiatry Psychiatr Epidemiol.

[B29] Darke S, Ross J, Williamson A, Mills KL, Havard A, Teesson M (2007). Patterns and correlates of attempted suicide by heroin users over a 3-year period: findings from the Australian treatment outcome study. Drug Alcohol Depend.

[B30] Kessler RC, McGonagle KA, Zhao S, Nelson CB, Hughes M, Eshleman S, Wittchen HU, Kendler KS (1994). Lifetime and 12-month prevalence of DSM-III-R psychiatric disorders in the United States. Results from the National Comorbidity Survey. Archives of general psychiatry.

[B31] Regier DA, Farmer ME, Rae DS, Locke BZ, Keith SJ, Judd LL, Goodwin FK (1990). Comorbidity of mental disorders with alcohol and other drug abuse. Results from the Epidemiologic Catchment Area (ECA) Study. Jama.

[B32] Teesson M, Havard A, Fairbairn S, Ross J, Lynskey M, Darke S (2005). Depression among entrants to treatment for heroin dependence in the Australian Treatment Outcome Study (ATOS): prevalence, correlates and treatment seeking. Drug Alcohol Depend.

[B33] von Limbeek J, Wouters L, Kaplan CD, Geerlings PJ, von Alem V (1992). Prevalence of psychopathology in drug-addicted Dutch. Journal of substance abuse treatment.

[B34] Gargiulo M, Waal HHE (2003). Maintenance in Seriously ill Addicts. Maintenance Treatment of Heroin Addiction.

[B35] Popova S, Rehm J, Patra J, Baliunas D, Taylor B (2007). Illegal drug-attributable morbidity in Canada 2002. Drug Alcohol Rev.

[B36] Connock M, Juarez-Garcia A, Jowett S, Frew E, Liu Z, Taylor RJ, Fry-Smith A, Day E, Lintzeris N, Roberts T, Burls A, Taylor RS (2007). Methadone and buprenorphine for the management of opioid dependence: a systematic review and economic evaluation. Health technology assessment (Winchester, England).

[B37] Darke S, Ross J, Teesson M (2007). The Australian Treatment Outcome Study (ATOS): what have we learnt about treatment for heroin dependence?. Drug Alcohol Rev.

[B38] Kornor H, Bjorndal A, Welle-Strand G (2006). Pharmacological therapies for opiate dependence, Systematic Review. Medikamentell behandling av opiatavhengighet.

[B39] Waal H (2007). Merits and problems in high-threshold methadone maintenance treatment. Evaluation of medication-assisted rehabilitation in norway 1998-2004. European addiction research.

[B40] Gossop M, Waal HHE (2003). Randomised and Controlled, but Irrelevant?. Maintenance Treatment and Heroin Addiction Evidence at the Crossroads.

[B41] Teesson M, Ross J, Darke S, Lynskey M, Ali R, Ritter A, Cooke R (2006). One year outcomes for heroin dependence: findings from the Australian Treatment Outcome Study (ATOS). Drug Alcohol Depend.

[B42] Fischer B, Rehm J, Kim G, Kirst M (2005). Eyes wide shut?--A conceptual and empirical critique of methadone maintenance treatment. European addiction research.

[B43] Bretteville-Jensen A, Amundsen EJ (2006). Omfang av sprøytemisbruk i Norge. SIRUS-rapport.

[B44] Waal H, Clausen T, Aamodt C, Lillevold PH (2006). LAR i Norge - Statusrapport 2005.

[B45] (2006). 2006 NATIONAL REPORT (2005 data) TO THE EMCDDA - NORWAY.

[B46] (2007). Hiv-situasjonen i Norge pr.  31. desember 2006. MSIS-rapport.

[B47] (2007). Årsrapport 2006 (annual report) - Meldesystem for infeksjonssykdommer.

[B48] Egeland A (2003). Presentation, 7th International Hepatitis C Conference, Edinburgh.

[B49] Kielland K Personer med samtidig alvorlig psykisk lidelse og omfattende rusmisbruk. Oslo, Norwegian Board of Health Supervision, 2000.

[B50] Skeie I, Brekke M, Lindbaek M, Waal H (2007). [General practitioners can take responsibility for medication-based rehabilitation]. Tidsskrift for den Norske laegeforening.

[B51] World Health Organisation International Classification of Diseases (ICD) - 10.

[B52] WONCA International Classification of Primary Care.

